# Combination of Whole Genome Sequencing and Metagenomics for Microbiological Diagnostics

**DOI:** 10.3390/ijms23179834

**Published:** 2022-08-30

**Authors:** Srinithi Purushothaman, Marco Meola, Adrian Egli

**Affiliations:** 1 Applied Microbiology Research, Department of Biomedicine, University of Basel, 4031 Basel, Switzerland; 2 Institute of Medical Microbiology, University of Zurich, 8006 Zurich, Switzerland; 3 Swiss Institute of Bioinformatics, University of Basel, 4031 Basel, Switzerland; 4 Clinical Bacteriology and Mycology, University Hospital Basel, 4031 Basel, Switzerland

**Keywords:** whole genome sequencing, metagenomics, epidemiology, surveillance, antimicrobial resistance, transmission, database, bioinformatics, combination

## Abstract

Whole genome sequencing (WGS) provides the highest resolution for genome-based species identification and can provide insight into the antimicrobial resistance and virulence potential of a single microbiological isolate during the diagnostic process. In contrast, metagenomic sequencing allows the analysis of DNA segments from multiple microorganisms within a community, either using an amplicon- or shotgun-based approach. However, WGS and shotgun metagenomic data are rarely combined, although such an approach may generate additive or synergistic information, critical for, e.g., patient management, infection control, and pathogen surveillance. To produce a combined workflow with actionable outputs, we need to understand the pre-to-post analytical process of both technologies. This will require specific databases storing interlinked sequencing and metadata, and also involves customized bioinformatic analytical pipelines. This review article will provide an overview of the critical steps and potential clinical application of combining WGS and metagenomics together for microbiological diagnosis.

## 1. Introduction

Three questions guide diagnostics in clinical microbiology: (i) Species identification: “who is there?”, (ii) biological functionality: “what are microorganisms doing?”, and (iii) interaction between microorganisms and the host: “are they linked?” [1]. These questions allow the assessment of different clinical scenarios using specific culture- or genome-based technologies. Culture-based microbiological diagnostic assays usually provide reliable species identification and allow the detection of mixed infections on agar plates. Culture also delivers established phenotypic readouts such as antibiotic susceptibility [2]. However, important shortcomings include the following: (i) Most phenotypic methods are time-consuming, requiring up to 72 h to obtain results [3,4]; (ii) many microbes are fastidious or non-culturable, thereby remain undetected, introducing a diagnostic bias [5]; (iii) information on virulence is often scarce due to a lack of standardized phenotypic readouts; and (iv) phenotypes for antimicrobial resistance profiling can be influenced by numerous parameters, e.g., type of agar plates, age of colonies, and environmental factors such as temperature and pH [6]. Genome-based diagnostics may help to overcome some of these limitations.

Since the first fully sequenced bacterial genome of *Haemophilus influenzae* type B became available in 1995 [7], sequencing technologies have rapidly evolved and are now used in patient care and infection control management [8]. Whole genome sequencing (WGS) and metagenomics use different approaches to determine the genomic content in a sample. WGS aims to analyze the whole genome of a single bacterial colony, while amplicon-based marker gene sequencing (e.g., 16S/ITS) or shotgun metagenomics focuses on microbial communities within a sample, usually without culture [9,10]. The costs per sequenced nucleotide have substantially decreased during the past decade [11,12] due to the expansion of sequencing capacities, the development of cost-effective technologies, advances in laboratory automation, and the progression of standardized workflows [13,14]. Today, knowledge and utilization have increased to a point where WGS can be applied in clinical microbiological diagnostics and surveillance not only in high-resource laboratories, but also in limited-resource environments [15,16,17].

Successfully linking the genotype to the phenotype for clinical applications requires a profound understanding of the diagnostic process, for example, when and how linking both types of information is appropriate. Sequencing capacity was further boosted during the COVID-19 pandemic [10,18], with more than 13.9 million SARS-CoV-2 genomes sequenced and made publicly available (www.covid19dataportal.org; accessed on 9 August 2022). This massive sequencing effort also resulted in a high degree of standardized analytical protocols and increased awareness of quality control of the sequencing data [19].

**Figure 1:**
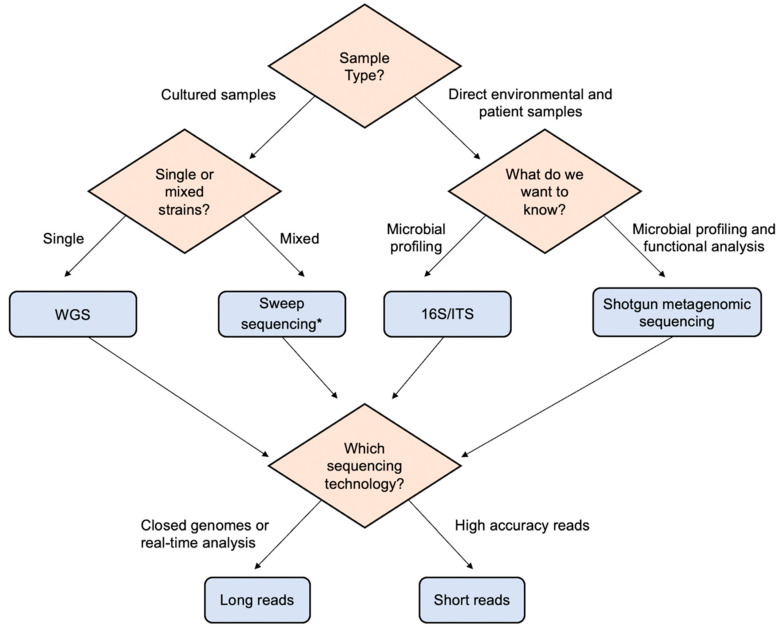
Decision tree for the selection of sequencing technology. The type of samples and research questions guide the selection of suitable sequencing strategies and sequencing platforms. The diamonds represent the checkpoints/questions, and the blue rectangles represent the sequencing strategies. * Sweep sequencing is a technique in which concurrent sequencing of multiple colonies at the same time is possible—this could also be considered a metagenomic approach.

The choice of the sequencing approach (WGS vs. metagenomics) is dependent on the clinical question and demand, e.g., rapid result, need for acute management vs. high-resolution result for typing. In addition, the available sample type influences the choice of technology. Figure 1 provides a decision flow chart for selecting the suitable sequencing technology.

With increasing numbers of available WGS and metagenomic datasets, the question arises as to whether a combination of both potentiates the technical resolution and the information produced for disentangling the microbial communities present in various environments and their transmission between them. While WGS still largely generates data from isolates grown on plates, culture-independent 16S metagenomics drastically reduces the time required for species identification, thanks to the more rapid and direct marker-gene sequencing and available high-quality databases. In the case of pathogens, WGS can elucidate risk potentials such as strain relatedness for outbreak detection, as well as the presence of genes potentially encoding antimicrobial resistance (AMR). Shotgun metagenomics can combine the advantages of both these methods and also shed light on the pathogens circulating in the environment. However, this comes with high costs, low standardization, and limited sequencing depth, often resulting in fragmented information from single bacterial genomes [20], and challenging bioinformatics hampers its application in clinics so far. A successful combination may allow the application of new statistical approaches such as data mining and enable the exploration of individual pathogens and host–pathogen interactions, as well as the complex interplay of microbial communities. This could pave the way for a more personalized risk assessment of colonizing or virulent pathogens and the transmission dynamics of microbes between different environments. How could a combined approach be applied to healthcare? What are the technical and analytical requirements? What are the potential use cases?

In this review, we will try to answer and discuss these critical questions.

## 2. Focus on Individual Microorganisms

In bacteriology and mycology routine diagnostics, WGS is most commonly applied to single bacterial isolates [21]. Nowadays, with the help of advanced molecular biology techniques, difficult-to-culture microorganisms can also be sequenced using WGS. For example, the sequencing of *Mycobacterium tuberculosis* from liquid media using the Mycobacteria Growth Indicator Tube (MGIT) [22] has been established, although with a slower turnaround time compared to targeted DNA enrichment directly from sputum [23]. Viruses or intracellular bacteria can be amplified or enriched using (i) pathogen-specific PCRs (e.g., for SARS-CoV-2 or Influenza) [24,25] or (ii) bead-based DNA pull-down assays, e.g., for *Chlamydia trachomatis* [26], human papillomaviruses [27], or Noroviruses [28]. Sweeps from solid agar plates with multiple mixed species can also be used, as shown for the mGEMS and mSweep bioinformatic pipelines, which were validated with *Escherichia coli*, *Enterococcus faecalis,* and *Staphylococcus aureus* [29].

*Quality control.* After sequencing, the typical analysis workflow includes quality assessment of the sequenced data (raw fastq files), followed by preprocessing of the sequenced reads (adapter trimming and filtering low-quality reads) and assembly [30,31,32]. The quality control (QC) monitors the following critical parameters: Read accuracy (Q30 score to measure the probability of incorrect base calling), genome coverage, genome completeness, and the number of contigs [33]. QC is followed by the assembly of the reads to generate contigs and possibly circularize the genome. Assembly is either performed de novo, against a reference genome map, or as a split k-mer analysis [34], which all may influence the subsequent typing resolution. Short-read and long-read sequencing can be used together to form hybrid assemblies, which allow the generation of high-quality genomes of single pathogens [35,36]. GC biases (GC-poor and GC-rich regions), which arise due to the genomic composition of the microorganisms, lead to uneven coverage during sequencing and might affect the resulting assemblies [37,38]. The library preparation techniques used for sequencing can also impact the assemblies [39,40]. In addition, contamination from different or even the same species needs to be excluded to ensure the reliability of the downstream analyses [41]. After assembly of the raw data, it is difficult to attribute the cause of errors. Specific QC pipelines such as AQUAMIS (Assembly-based Quality Assessment for Microbial Isolate Sequencing) allow for automating this process [42].

*Identification.* The assembled genomes are used to identify microbes at species or subspecies levels. Genomic-based species annotation of assembled genomes appears trivial at first but can have unexpected caveats for certain species, even for well-known pathogens [43,44]. The bacterial taxonomy is ruled by the International Code of Nomenclature of Prokaryotes (ICNP) under the supervision of the International Committee on Systematics of Prokaryotes (ICSP) based on different phylogenomic approaches [45]. The extent of sequencing and the consequent possibility for a higher-resolution distinction between the genus and species have sparked new discussions on a revision of the ICNP [46,47]. Therefore, a crucial aspect is correct annotation with well-curated and internationally accepted databases to reliably identify a bacterial species.

The Type Strain Genome Server (TYGS, (https://www.dsmz.de/services/online-tools/tygs, accessed on 31 May 2022) is a particularly well-curated database, and the accompanying software offers the possibility to identify potential new bacterial species [48]. The GTDB (Genome Taxonomy Database) is another highly curated, phylogenetically consistent, and genome-based taxonomy database for annotation, backed by GTDB-Tk software for genome annotations [49]. Despite these well-curated databases, identifying a bacterial species from genomic data can still be challenging. For example, currently available software tools such as PubMLST, MetaPhlAn3, and Mykrobe-predictor showed variable performances in correctly identifying non-tuberculous mycobacterial species. Sensitivities ranged from 57–100% and specificities from 83–98%, which could be attributed to the different databases and algorithms used [50].

*Molecular epidemiology.* WGS has become the reference standard for microbial typing to address epidemiological questions. Increasingly standardized workflows and quality management have been established [51,52]. Most studies use genome comparison to a reference (mapping) or within-cluster mapping with either core genome (cg) multi-locus sequence typing (MLST), SNP-based comparison, or more recently, also split k-mer analysis (SKA) [53,54,55,56]. Pairwise genome comparisons using SKA showed a higher resolution compared to cgMLST of *Enterococcus faecium* [34]. Outbreak investigation and transmission studies benefit from large publicly available genome datasets to compare potentially related strains with non-outbreak-associated isolates. As an example, the NCBI pathogen browser covers a selection of 40 bacterial and fungal species with more than 1 million available genomes (accessed on 31 May 2022; [57]). Similarly, the Swiss Pathogen Surveillance Platform (www.SPSP.ch; accessed on 31 May 2022 [58]), the Eukaryotic Pathogen, Vector, and Host Informatics Resource (VEuPathDB) (https://veupathdb.org/veupathdb/app/static-content/about.html, accessed on 4 August 2022), or the European Nucleotide Archive (https://www.ebi.ac.uk/ena, accessed on 31 May 2022) contain genomic data on viral, fungal, and bacterial pathogens, which allow epidemiological studies with important epidemiological metadata. Outbreak analyses in hospitals often focus on antibiotic-resistant pathogens, and the value of WGS has been documented in many instances for the transmission of bacterial strains [59,60,61,62,63] or plasmids with multi-drug resistance genes [64].

*Inference of function.* Assembled genomes from WGS can be used to infer phenotypes, such as AMR and virulence [65,66]. Curated databases such as ResFinder [67] and CARD [68] are commonly used for the detection of AMR genes. The combination of highly curated databases and underlying algorithms plays a major role in prediction accuracy [69,70]. For example, the concordance of Mykrobe-based (https://www.mykrobe.com, accessed on 31 May 2022) AMR prediction with phenotypic testing was 94% in *Mycobacterium tuberculosis* compared to TB-profiler, MTBseq, and other benchmarked tools [71]. Single-nucleotide polymorphisms (SNPs) can be associated with a loss or gain of function. For instance, SNPs were conclusively linked to functional impairment of porins within the species *P. aeruginosa* (*oprD;* [72]) and de-regulating feedback loops affecting transcription factors were linked to beta-lactamase expression (*ampR and ampC;* [73,74]).

A promising new approach for identifying specific functional links is Genome-Wide Association Studies (GWAS), which have recently been translated from human genetics to microbiology [75,76]. A GWAS identifies genes, k-mers, and/or SNPs enriched in microorganisms linked to a particular phenotype. Examples include daptomycin resistance in *Staphylococcus aureus* linked to mutations in *mprF* [77], and clinical phenotypes such as invasiveness following urinary tract infection linked to the adherence factor *papGII* of *E. coli* [78,79]. Newly developed bioinformatic GWAS pipelines such as PowerBacGWAS provide power calculations to determine statistically significant sample size association testing [80]. Clinical validation of the identified genetic markers in randomized controlled trials, similar to any other clinically used biomarker, is necessary to assess their clinical value. In the next few years, we expect that more bacterial genetic markers will lead to diagnostic applications, e.g., virulence assessment or AMR surveillance [81,82,83].

## 3. Focus on Bacterial Communities

When sequencing bacterial communities, two metagenomic sequencing approaches can be distinguished, amplicon-based and shotgun metagenomics. The former targets a marker-gene or a segment thereof, which allows for the resolution of the bacterial community structure. The latter sequences/covers representative genetic material of a specimen, usually including the DNA of the host.

*Identification with amplicon-based sequencing.* In recent years, amplicon-based short-read sequencing of marker genes, in particular 16S rRNA gene (16S) and its variable regions (V1-V9), became highly popular for explorative studies in ecology research. Alternative universal marker genes include the bacterial *rpoB* or the fungal internal transcribed spacer 1/2 (ITS-1/2) [84]. 16S sequencing has also been applied for clinical diagnostics, e.g., in abscesses [85], urinary tract infections [85,86], or sepsis [87], as well as for environmental studies. Oberaune et al. profiled the microbiome of intensive care units (ICUs) and found higher microbial diversity compared to culture-dependent techniques [88].

For taxonomic profiling, the 16S sequence reads are assigned to representative sequences, such as operational taxonomic units (OTUs) through clustering [89] or to amplicon sequence variances (ASVs) through a denoising algorithm [90,91,92]. There are various OTU clustering algorithms available [93,94,95]. Imprecisely clustered sequences can give rise to inaccurate OTU classifications, which has a significant impact on downstream analyses. Therefore, alternatives for OTUs have been suggested, such as pairwise alignment sequence dissimilarity (PSD), MSA-based sequence dissimilarity (MSD), and phylogenetic branch length distance (BLD) [96].

For species identification, single variable regions are usually not suitable [97], and some variable regions such as V7 are known to yield ambiguous identifications [98]. However, bioinformatics tools allow the combination of individually sequenced variable regions from one strain, thereby delivering species-level resolution within samples [99,100]. A recent promising approach uses 16S-23S de novo assembled sequencing data and a Basic Local Alignment Search Tool (BLAST) approach with a newly developed database for species identification [101]. Recently, long-read-based full rRNA operon region analysis has also been described, providing an even higher resolution [102].

*Inference of function with amplicon-based sequencing.* Amplicon-based sequencing lacks the possibility to directly study functional aspects of the species within a sample. However, metabolic inference approaches such as Paprica, Picrust2 [103,104], and Tax4Fun2 [105] are available. These tools use hidden-state prediction (HSP) algorithms, which allow the estimation of metabolic functions based on representative genomic content from a well-described bacterial community. A key problem in using such databases is the relatively low correlation between the relative abundance of their specific functions [106] and the high population variability, limiting these tools in applications with defined cohorts.

*Identification with shotgun metagenomic sequencing.* Shotgun metagenomics facilitates untargeted sequencing of all microbial genomes present in the sample [107]. The dataset generated is much more complex than amplicon-based sequencing [108]. Standardization of the methodological and analytical workflows has just started [109,110]. Proof-of-concept studies have shown the potential clinical impact in pathogen identification within culture-negative samples of, e.g., meningitis and encephalitis [111], sepsis [112,113,114], pneumonia [115,116], and prosthetic joint infections [117,118]. The detection limit of shotgun metagenomics is affected by slow-growing microorganisms or if the potential pathogen is present in low abundance. Different protocols to increase the sensitivity have been developed, e.g., a short, specific culture step for certain pathogens [117]. However, in primary non-sterile body sites, this may introduce a critical diagnostic bias. The performance in bacterial detection and identification was compared between shotgun metagenomics and 16S amplicon-based sequencing, where shotgun metagenomics showed a slightly higher sensitivity (46.3% vs. 38.8%) than 16S [119]. Similarly, Gu et al. compared shotgun metagenomic sequencing using Illumina (short-read) and Nanopore sequencing (long-read) platforms for pathogen identification and validated the results with traditional culture-based methods and also with 16S and 28S-ITS PCRs for bacterial and fungal species. They have also shown that the real-time analysis offered by Nanopore sequencing enabled a reduced turnaround time for pathogen identification [120]. The latest add-on feature with the ONT sequencing platform is “adaptive sampling”, which allows for enriching or depleting sequenced DNA from selected species selectively in a software-controlled manner during sequencing [120,121]. This is useful for clinical samples such as body fluids and swabs where human DNA largely outweighs non-human DNA, and the depletion of host DNA consequently increases the pathogen detection sensitivity [122,123]. On the bioinformatics front, new software tools such as SMAGLinker, Strainberry, and STrain Resolution ON assembly Graphs (STRONG) allow for obtaining strain-resolved genomes in microbiota samples for both short-read and long-read sequencing data. Metagenomic sequences are assigned to several bins and merged for taxonomic identification [124,125,126]. For RNA viruses, the RNA-dependent RNA polymerase (RdRp) can be used as a baseline core motif for species identification [127,128].

*Inference of function with shotgun metagenomics.* Deciphering microbiota functions is crucial for predicting clinical phenotypes. The generation of high-quality metagenomically assembled genomes (MAGs) from metagenomic datasets facilitates the understanding of microbial ecosystems by elucidating detailed metabolic pathways and horizontal gene transfer networks [124]. The prediction of function requires various bioinformatic tools and databases, of which dozens have been developed [129,130,131]. Since not all the genes and annotations are known yet, the databases for functional annotation (e.g., KEGG and EggNOG) are still incomplete, and thus not all metabolic functions can be inferred from the MAGs. An important aspect of functional inference is the detection of AMR genes. Knowledge about local microbiome compositions and hotspots of AMR genes present in the environment may potentially be used to trigger further investigations. Chng et al. sequenced environmental surface swabs collected from a hospital and combined short- and long-read sequencing to determine distinct ecological niches present in various regions of the hospital [132]. AMR monitoring from sewage samples is an interesting surveillance tool [133], which is not only used for pathogen surveillance, e.g., SARS-CoV-2 [134], but also for virulence monitoring [135]. Perry et al. compared sewage samples from a hospital and the surrounding community sewage plants. The authors modeled the correlation between antimicrobial usage in the hospital and resistance gene abundances and showed that sewage from the hospital has a higher percentage of Antibiotic Resistance Genes (ARGs) compared to the communal sewage samples [136]. Another interesting function to study would be the microbial association network from microbiome data in order to capture the interactions between the various identified species. The microbial association catalog (mako) is one such graph-based database compiled from 60 microbiome studies, which allows for a user-friendly network motif search [137] to infer the associations within the microbiome. The next step is to link more complex microbiological communities, on the species level or the genetic content level, to particular clinical phenotypes. A microbiome-wide association study [138,139,140,141] could help to identify crucial networks of communities and link this to, e.g., treatment response in mixed infections, the risk of colonization, or invasive infection [142]. Another interesting application of the interaction of microbiota or microbiome would be immune modulation in cancer, where specific species are enriched or show immunomodulatory effects for check-point inhibitors during cancer therapy [143,144,145,146,147].

## 4. An Integrated Approach of WGS and Metagenomic Sequencing

Comparing the currently available WGS and metagenomic technologies and approaches (refer to Table 1 for the possibilities and limitations) to study microbial features, it becomes clear that no single approach can answer all diagnostic or research questions. Both approaches show potential for complementary usage and data analysis, which may potentiate the output and provide novel insights into host–pathogen interactions, clinical outcomes, and pathogen surveillance in various environments. However, to achieve this goal, we first need to link the sequencing outputs. In a prospectively built database, e.g., during surveillance or monitoring of a patient, a unique identifier could be used to merge one or more WGS datasets with a metagenomic dataset. Ideally, additional clinical, microbiological, or epidemiological metadata would be added, such as the time and space of acquisition of each dataset and potential phenotypic readouts such as AMR or metabolic profiles. The below section discusses the data formats, clinical use cases, bioinformatics tools, and the quality control pointers for combining WGS and shotgun metagenomics sequencing data.

*Examples ofcombined WGS and shotgun metagenomics approaches.* A combined, integrative approach allows one to look for similarities in the dataset and is the primary aspect of merging both data sources. Such a combination may be used for identification, functional readouts, and typing. The ability to quantify and detect bacterial strains within heterogeneous environments has applications in numerous fields including diagnostics [148], clinical studies for the microbiome [149], bio surveillance, One Health [150,151], outbreak investigations [150,152,153], providing insight into the spread of antibiotic resistance [152], tracking the progression of within-host bacterial evolution [153], and exploring diverse environments [154,155].

Such an integrated approach was used to investigate an outbreak of carbapenemase-producing *Enterobacter hormaechei*. The source of the strain could be allocated to the plumbing and water resources in a hospital. The combination of short- and long-read sequencing enabled the resolution of the complete plasmid of the resistance gene carrier (IMP-4) and thereby the monitoring of its transmission across the hospital environment [156]. Similarly, an outbreak of a carbapenem-resistant *Acinetobacter baumannii* was investigated and the source was again linked to the plumbing system of a hospital [157]. This outbreak investigation resulted in an internal database of circulating pathogens in the environment, and it allowed one to restrict the transmission of the resistant strain and provided information about the recurrence of the pathogens in the hospital wards.

A new addition to metagenome sequencing is the implementation of single-cell bacterial sequencing technology, which combines shotgun metagenomics and WGS for strain resolution and allows the tracking of mobile elements. For example, Zheng et al. devised Microbe-seq, single-microbe genomics to achieve sub-species resolution from the human gut microbiome. Using microfluidics, they have captured single microbes in liquid droplets, lysed the cells, and barcoded the DNA followed by whole genome amplification. Computationally, the authors have co-assembled single-amplified genomes (SAGs), recovered from the whole genome amplification of single bacterial cells, and characterized the horizontal gene transfers within the strains of the same species [158]. This approach therefore allows one to study the transfer of AMR or virulence genes. One hypothetical application for combining WGS and shotgun metagenomics data could be as follows: We can consider the identified microbiome from the shotgun metagenomic sequencing as a restricted database of microorganisms present in a clinically relevant environment. The assembled genome of a particular pathogen obtained from WGS can then be used as a query sequence, which can be searched against this database (and vice versa). During a hospital outbreak investigation, for example, this would allow the quick identification of the outbreak source, assuming environmental screening is performed at regular intervals and enriched with useful metadata. In other words, the pathogen can be traced back to an environmental origin in the hospital if a hit of high similarity is found in the database, while it is likely an introduction from outside if it cannot. This approach could also be applied for tracking the transmission of mobile genetic elements across environments. On the community front, the microbiome data obtained could also be used for identifying at-risk populations based on microbial distributions. One major drawback of such a combination is the added cost of regularly sequencing the environment of interest.

*Data readouts.* Typically, short-read sequencing data from Illumina machines and long-read sequencing data from Nanopore and PacBio result in fastq files after base calling from their respective sequencing data formats. Contigs, assembled genomes, or segments are usually in FASTA format. As a consequence, merging data from either platform is computationally convenient.

*Possible computational methods for strain-level microbial detection from WGS and shotgun metagenomic sequencing.* An inter-linked dataset allows the use of the contained data as a reference for mapping. A range of bioinformatics pipelines and methods for strain-level microbial detection in metagenome sequencing data have been developed [159]. In principle, methods are based on (i) assembly-based reconstruction and (ii) methods operating with or without a reference database. We are only at the beginning of using WGS and metagenomics in clinical settings, therefore it is crucial to have a comprehensive benchmark across different (clinical) applications to validate performances and standardize the available tools.

Assembly-based approaches identify single strains in mixed reads by whole genome assembly. Sufficient differences in the genomes are necessary to separate or cluster, e.g., bacterial variants into distinct strains [160]. This approach requires sufficient read length and sequencing depth to reach at least one variant site in most reads. Tools such as EVORhA, STRONG, StrainGE, and Strainberry deconvolute the assemblies from short- and long-reads to provide strain-level resolution [125,126,153]. Hypothetically, this offers interesting applications in AMR surveillance, as these strains could then be compared using a curated database containing local endemic AMR-relevant strains. The assembly step in WGS can also be combined with a metagenomic dataset, which allows the identification of specific single strains within a bacterial community [161]. Zlitni et al. performed short-read and read cloud metagenomic sequencing together with metatranscriptomics to monitor the sub-strain populations within a patient’s gut [162]. Meanwhile, Ivanova et al. used shotgun metagenomics with chromosome conformation capture (Hi-C) technology. This resulted in high-quality MAGs together with plasmids, as the technology also linked reads between genome and mobile genetic elements [163]. These approaches allow for identifying specific pathogens or plasmids harboring multi-drug resistance in a specific environment.

Furthermore, full genome alignment-based methods allow strain classification by aligning reads directly to a selection of reference genomes and applying stochastic models to calculate the likelihood of association between a specific read and reference [164]. Pathoscope [165] is a classification pipeline using different aligners, including GNUMAP [166], Bowtie 2 [167], and BLAST [168], and scores for each alignment reflect the likelihood that the read source matches the reference assembly. Furthermore, a semi-quantitative assessment can be reached for strain abundance based on the number of reads mapping to each reference. Alignment-based detection works within clear and well-separated sub-lineages. However, the reference database is critical in closely related strains.

Substantial computing time can be saved by aligning a set of genetic markers, rather than the complete genome. These marker-based methods classify genetic diversity within a sample using a database of, e.g., unique genes [169], SNPs, or k-mers [170]. Pattern-based methods also require a reference database for statistical models. However, pattern-based methods initially pre-process extracted features and use these features for a new classifier algorithm, which results in substantially decreased analytical time. MIDAS is one such bioinformatic tool, applying this concept for species and strain-level taxonomic identification [171]. The k-mer-based tool GSMer identifies strains by using a strain-specific database of strain-specific k-mers, or genome-specific markers (GSM) [172]. In this tool, each strain is represented by at least 50 GSMs, and strains with less than 50 unique GSMs are excluded. Strains are only identified if there is perfect alignment for all 50 GSMs, resulting in high specificity, but potentially low sensitivity. Such an approach may be rather useful for clonal, slowly evolving strains without high rates of genetic adaptation.

*Quality control.* There are several quality factors to consider, before integrating the data from different omics techniques: (i) Regular update of databases: This not only includes technical and software updates but also includes the epidemiological content covered by the database, e.g., via regular shotgun metagenomic sequencing of a given environment; (ii) sequencing depth and coverage: Care should be taken to assess the quality of the data obtained from each of these omics technologies. Since the performance of these technologies is prone to bias, standardized workflows will result in reproducible read cut-off values for depth and coverage.

## 5. Conclusions and Future Trends

With the use of clinical WGS and metagenomics on the rise, in part due to the SARS-CoV-2 pandemic, environmental screening for microbes has become feasible and cost-effective [151]. Researchers have combined various sequencing platforms in proof-of-concept studies for pathogen identification, the characterization of virulence and resistance genes, and the typing of relatedness between bacteria, viruses, and fungi. Indeed, mapping the ecological niches of the pathogens, e.g., in the hospital [156,157] as well as the environment may potentiate the effects. It has also been shown that it is possible to integrate 16S and shotgun metagenomics for microbiome studies, where the expected readouts are taxonomic abundance, diversity, and functional annotations. While 16S can provide the identified taxonomy, shotgun metagenomic sequencing can validate it with the genomes of the identified microbes and their functional annotations [173,174,175,176]. Harmonization and standardization of the individual technologies will likely also move forward. For clinical applications, clear, controlled, and reproducible protocols are necessary, reflected in the regulatory requirements such as the recently established In Vitro Diagnostic Regulation (IVDR).

So far, only a few studies have shown how to successfully integrate and use the additive and potential synergistic effects of both technologies. One limitation is the need for standardization and harmonization between protocols and workflows, and the added sequencing costs of two technologies being applied along with the costs linked to data storage and maintaining the bioinformatic pipelines and databases. Metadata with a sufficient spatio-temporal resolution (e.g., sample isolation date and geographical location) and additional epidemiological context may become important to use this potential. The access to metadata goes hand in hand with the FAIR data-sharing principles (Findable, Accessible, Interoperable, and Reusable), and the need for databases to allow for properly annotating the interlinked status between omics data types. Overall, combining WGS and shotgun metagenomics brings out complementary benefits by incorporating missing pieces of information.

## Figures and Tables

**Table 1:** Comparison of whole-genome sequencing, marker gene-based amplicon sequencing, and shotgun metagenomic sequencing. GWAS = Genome-Wide Association Studies. SNP = Single Nucleotide Polymorphism. The symbol “$” represents the cost of sequencing. Higher number of $ = higher cost. The symbol “+” represents the turnaround time for the sequencing strategies. Higher number of + = longer turnaround time.

Parameters	WGS	16S/ITS	Shotgun Metagenomic Sequencing
Sample	Cultured or enriched microorganisms	Swabs from body sites, stool samples, body fluids or tissue samples, and sewage	Swabs from body sites, stool samples, body fluids or tissue samples fecal matter, and sewage
Species identification	Yes	Yes	Yes
Degree of resolution	Species-Strain level	Genus-Species level	Species-Strain level
Complete genome	Complete genome possible depending on sequencing platforms	No	Near complete to gapped genomes.
SNP analysis	Yes	No	Yes
GWAS	Yes	No	Yes
Identification of virulence factors and resistance genes	Yes	No	Yes
Microbial community profiling	No	Yes	Yes
Cost	$$	$	$$$
Turnaround Time (TAT)	+	++	+++

## Data Availability

Not applicable.
